# Cultural adaptations and methodological innovations to group model building for the systems actions to reduce malnutrition in all its forms in Southeast Asian countries and China (SYSTAM CHINA-SEACS International Consortium) project

**DOI:** 10.1186/s12966-023-01510-5

**Published:** 2023-09-18

**Authors:** Bai Li, Zouyan He, Remco Peters, Steven Allender, Yunfeng Zou, Weiwen Zhou, Jianfeng Lao, Bee Koon Poh, Boyd Swinburn

**Affiliations:** 1 Centre for Exercise, Nutrition and Health Sciences, School for Policy Studies, University of Bristol, Bristol, UK; 2 School of Public Health, Guangxi Medical University, Nanning, Guangxi China; 3 Global Centre for Preventive Health and Nutrition (GLOBE), Institute for Health Transformation, Faculty of Health, Deakin University, Geelong, VIC Australia; 4 Institute of Nutrition and School Health, Guangxi Zhuang Autonomous Region Center for Disease Control and Prevention, Nanning, Guangxi China; 5 Fang Cheng Gang Health Commission, Fangchenggang, Guangxi China; 6 Centre for Community Health Studies (ReaCH), Faculty of Health Sciences, Universiti Kebangsaan Malaysia, Kuala Lumpur, Malaysia; 7 School of Population Health, University of Auckland, Auckland, New Zealand

**Keywords:** Malnutrition, Systems Approach, Group Model Building, Participatory, Co-production, Policy Engagement, China, Asia, Obesity, Intervention development

## Abstract

**Background:**

Group Model Building (GMB) is a participatory system dynamics method increasingly used to address complex public health issues like obesity. GMB represents a set of well-defined steps to engage key stakeholders to identify shared drivers and solutions of a given problem. However, GMB has not yet been applied specifically to develop multi-duty interventions that address multiple inter-related issues such as malnutrition in all its forms (MIAIF). Moreover, a recent systematic review of empirical applications of a systems approach to developing obesity interventions found no published work from non-western, low- and middle-income countries (LMICs). In this paper we describe adaptations and innovations to a common GMB process to co-develop systemic MIAIF interventions with Chinese decision-makers.

**Methods:**

We developed, piloted and implemented multiple cultural adaptations and two methodological innovations to the commonly used GMB process in Fang Cheng Gang city, China. We included formal, ceremonial and policy maker engagement events before and between GMB workshops, and incorporated culturally tailored arrangements during participant recruitment (officials of the same seniority level joined the same workshop) and workshop activities (e.g., use of individual scoring activities and hand boards). We made changes to the commonly used GMB activities which enabled mapping of shared drivers of multiple health issues (in our case MIAIF) in a single causal loop diagram. We developed and used a ‘hybrid’ GMB format combining online and in person facilitation to reduce travel and associated climate impact.

**Results:**

Our innovative GMB process led to high engagement and support from decision-makers representing diverse governmental departments across the whole food systems. We co-identified and prioritised systemic drivers and intervention themes of MIAIF. The city government established an official Local Action Group for long-term, inter-departmental implementation, monitoring and evaluation of the co-developed interventions. The ‘hybrid’ GMB format enabled great interactions while reducing international travel and mitigating limitations of fully online GMB process.

**Conclusions:**

Cultural and methodological adaptations to the common GMB process for an Asian LMIC setting were successful. The ‘hybrid’ GMB format is feasible, cost-effective, and more environmentally friendly. These cultural adaptations could be considered for other Asian settings and beyond to address inter-related, complex issues such as MIAIF.

## Background

Malnutrition in all its forms (MIAIF) is the largest cause of disease and premature death globally, and in low- and middle-income countries (LMICs) [1]. MIAIF broadly includes undernutrition (e.g. underweight and micro-nutrient deficiencies) and obesity and diet-related non-communicable diseases (NCDs, such as type 2 diabetes). To tackle MIAIF in Asian LMICs effectively, coordinated interventions that target shared drivers of multiple forms of malnutrition across multiple components of the food systems are required [2]. Decision-makers who have the power to change local policy and regulations within or across various sections of the food systems need to be included in the development, implementation and evaluation of such interventions. This helps to ensure that the developed interventions recognise and support local contexts and goals, close the gap between research and practice, maximise impact and increase the likelihood of achieving long-term effects [3, 4].

Group Model Building (GMB) involves the use of a range of highly developed, rigorous methods from participatory community-system dynamics. System dynamics is one of several approaches within system science that involves identifying the factors of cause and effect and understanding their interrelationships within system structures and how they interact to drive complex problems and observed outcomes. In system dynamics, causal maps or models can be utilised to understand how different components within the system (e.g., food system), fit together, interrelate and change over time (feedback loops) produce undesirable nutrition outcomes [5, 6].

GMB allows for co-creation of these system logic models and have been used to underpin interventions to address complex issues. A set of protocols (also referred to as ‘scripts’) were developed to facilitate GMB activities [6] from the open access web source ‘Scriptapedia’ which was developed and used as a handbook for designing structured GMB sessions [6]. GMB may provide the means to engage local decision-makers across different sections of the food system to co-create a Causal Loop Diagram (CLD) which visualises the common, system-level drivers for MIAIF. This allows a diverse group of decision-makers to see how their work connects or contributes to the complex system, and to agree on the areas where they can act to change to the system [6]. This co-production process enables joint identification and prioritization of system-wide interventions to facilitate coordinated actions among its participants [4]. The co-creation has potential to maximise the impact and sustainability of the developed interventions by ensuring that the ownership of the interventions co-developed with the researchers rests with the decision-makers [7–11].

While this method has been used for co-developing systemic interventions to address single public health concerns such as obesity, and related unhealthy behaviours, its application to address MIAIF has not been studied and tested. GMB has already been applied to other health-related issues (e.g., health service, infectious disease and water use) in non-western, LMICs settings [12–15]. However, our recent global scoping review of empirical applications of a systems approach to obesity interventions found no published work from LMICs [16]. This raises the question as to whether this method is culturally acceptable, and practically feasible to co-develop multi-duty nutrition actions in these settings. Moreover, GMBs have evolved from a tradition of facilitating a process involving in-person events. For most public health projects that involve international partnerships, intercontinental flights occur frequently which is accompanied with increased environmental impacts [17]. Considering the environmental concerns and impracticalities of international travelling, in particular during public health crises, alternative formats needed to be considered. There is a growing body of literature indicating that a variety of online GMB formats are feasible [18, 19]. One example being adaptations due to COVID travel restrictions and the mix of in person and remote workshop delivery in a diabetes prevention project set in the remote Indian Ocean Territories [20]. Where combined in-person and fully online GMB workshops were implemented in response to COVID-19 lockdowns, a preceding study in United Kingdom (UK)/Canada settings adopted a hybrid design where participants and facilitators joined online alternately in otherwise in-person settings [19, 21, 22]. A ‘hybrid’ formats that allow for simultaneous online and in-person interactions may increase efficiency, and have the advantages of increasing participant engagement (both among the participants and with the GMB facilitation team) which can ultimately nurture inter-sectoral and long-term partnerships [18, 19]. The hybrid GMB format is still in its infancy, with limited detailed documentation on its design and processes. It is also unknown whether a hybrid GMB format would be feasible in China and other Asian LMIC settings.

To address the above issues, we purposively developed and tested a culturally relevant ‘hybrid’ format to run GMB workshops for developing systemic interventions for MIAIF in an Asian, developing country, with the intention that the developed methods can be applied or adapted in other LMICs in Asia and beyond. This study was conducted as part of a multi-nation research consortium entitled ‘Systems Actions to Reduce Malnutrition In All Its Forms in Southeast Asian Countries and China’ (SYSTAM CHINA-SEACS).

In this paper we describe multiple cultural adaptations and two innovations that we made to the common GMB process and standard script to successfully co-develop systemic MIAIF interventions with Chinese decision-makers. We will also discuss what we learnt from this process and its potential implications internationally.

## Methods

### Research setting and setting the boundaries

We developed, piloted and implemented the cultural adaptations and methodological innovations to the usual GMB process in the city of Fang Cheng Gang (FCG), Guangxi Zhuang Autonomous Region, China. Ethical approval was granted by University of Bristol’s Policy Studies Research Ethics Committee (Reference number: SPSREC/20–21/168) and Guangxi Medical University’s Medical Ethics Committee (Reference number: 20,210,164).

**Fig. 1:**
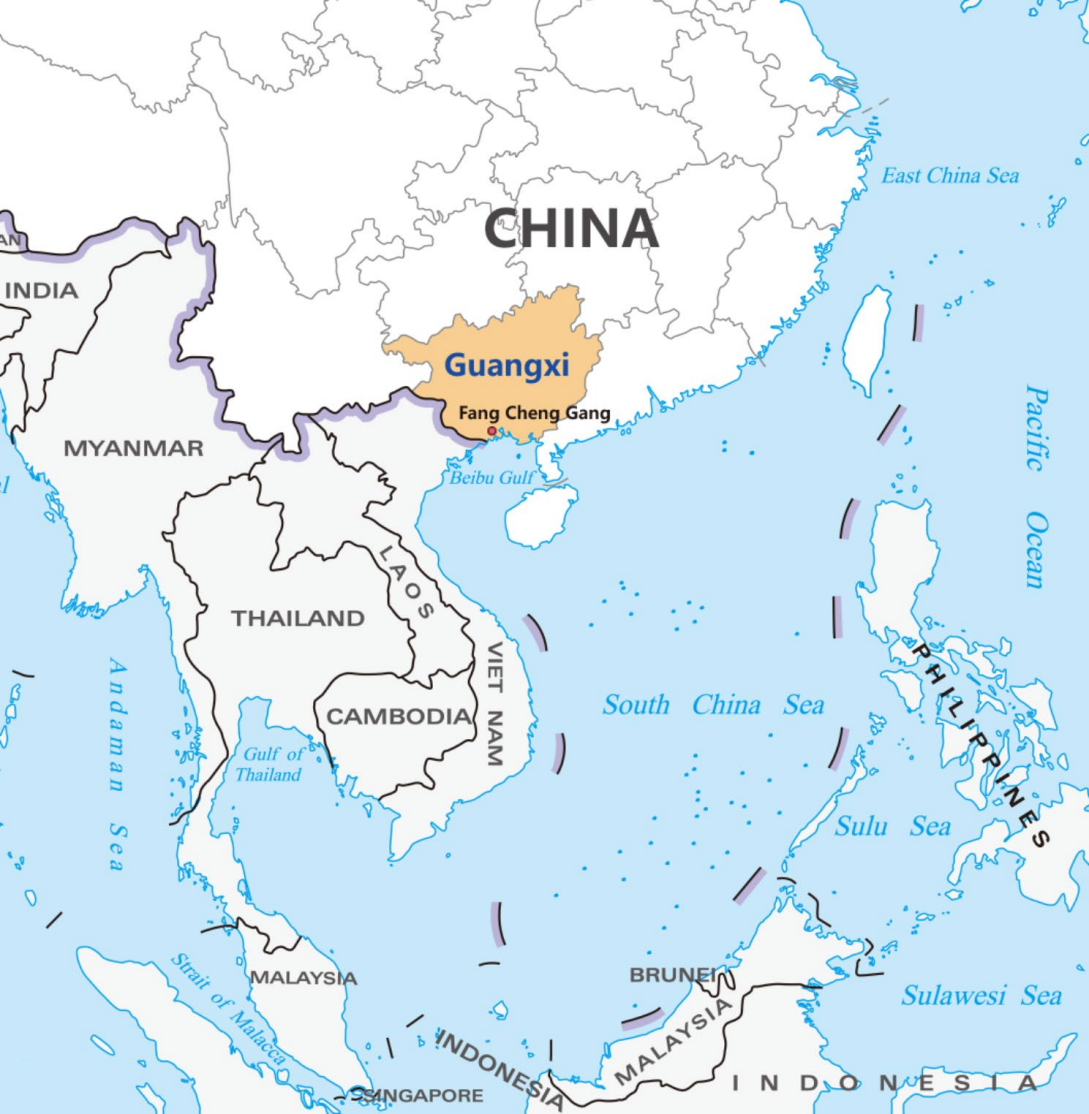
Geographic location of Guangxi Zhuang Autonomous Region and Fang Cheng Gang city

Guangxi Zhuang Autonomous Region is a province in south China, home to 57 million residents [23]. Similar to other low- and lower-middle income countries in SEA, MIAIF is prevalent in China, and specifically, the Guangxi province where overnutrition is still increasing while undernutrition remains a persistent significant public health concern [24, 25]. The research team initially set MIAIF as the problem scope with reference to the WHO definition for MIAIF, which was validated and agreed upon by the local health authority [26], specifically the Guangxi Health Commission (GXHC). Guangxi plays a significant role in promoting cooperation in public health and socio-economic development in SEA as it is neighbouring Association of Southeast Asian Nations (ASEAN) member countries (Fig. 1) and hosts the biennial China-ASEAN Health Forum. For these significant reasons, we selected Guangxi as the province for collaboration in the SYSTAM CHINA-SEACS.

FCG city with a population of one million, is a city in the south of Guangxi and the southwestern coastline of mainland China. Being a coastal city, FCG city has an oceanic climate and is a significant food producer (e.g., aquatic products) similar to the neighbouring SEA countries. FCG plays an important role in international cooperation in food safety, nutrition and public health in this region because it is the permanent host city for the biennial China-ASEAN Cooperation Forum on Food Safety, Nutrition and Health. Therefore, the research team and the provincial health authority (GXHC) jointly selected FCG as the pilot city for the SYSTAM CHINA-SEACS project. The FCG food system was specifically defined as the system boundary with reference to United Nations’ definition for food systems and through consultations and deliberations with key local health authorities [27]. Decision-makers from 22 governmental departments/bureaus and food businesses representing the entire local food systems (covering agriculture; environmental protection and climate change; food manufacturing, processing and packaging; food advertising; public media and leisure; transportation; retailing (food distribution); finance and budgeting; urban/economy development; food safety and regulation; food waste management; taxation; education sector; poverty and welfare; child and maternal care; public health/healthcare sector and so on) were invited to the GMB workshops and engaged in the co-creation of a CLD. In this, and many other Asian settings, involving decision-makers in the research project can promote a sense of project ownership among the decision-makers, maximising long-term support and impact [3, 4].

### The common GMB process

**Fig. 2:**
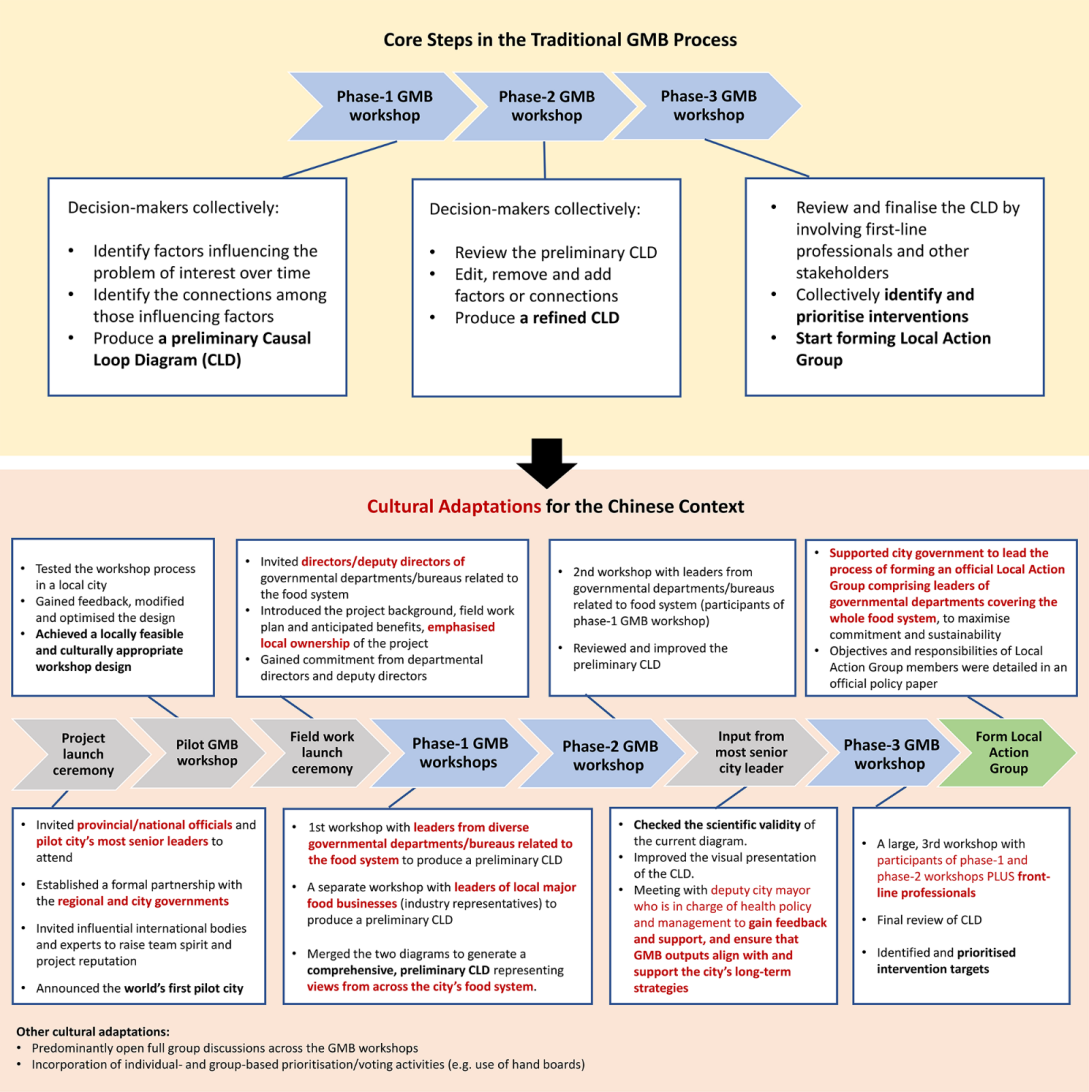
Procedural and cultural adaptations to the standard GMB process

A commonly used version of the GMB process consists of three phases (Fig. 2) which has been proven to be successful with obesity research in high-income countries [28, 29]. The phase-1 workshop encourages decision-makers to collectively identify factors influencing a complex problem and the connections among those factors, resulting in a preliminary CLD. The phase-2 workshop invites decision-makers to review and expand upon the initial CLD. In the phase-3 workshop, the CLD is further reviewed and refined. The decision-makers are also engaged to identify and prioritise interventions, and form local action groups for intervention implementation [6, 30]. In between the workshops the research team checks the scientific validity and improves the visualization of the CLD. While these represent a commonly used GMB process in obesity research, the phases and steps may differ depending on the circumstances and issues of interest [6].

### Cultural adaptations

We organised multiple additional events before and between the 3 common phases to maximise success and impact in a culturally novel setting. We also incorporated multiple culturally tailored arrangements during participant recruitment process and workshop activities. Figure 2 presents the adopted process.

#### Culturally tailored project engagement activities

Several activities were held to maximise local collaboration and commitment, and to create a sense of project ownership among local stakeholders. We held two formal launch ceremonies prior to the first workshop as ceremonial events are known to be important in the Chinese culture to announce and celebrate major initiatives. We organised the first official project launch ceremony to effectively consolidate our partnership with the regional (Guangxi province) health authority. We also invited national officials, the deputy mayor of FCG city who is responsible for public health policy, World Health Organization (WHO) Representative to China and international renowned experts to promote local support and raise the project’s local reputation.

To further strengthen local support, we organised a second project launch ceremony to establish our partnership with the Municipal People’s Government (MPG) of FCG which consists of the mayor and several deputy mayors who preside over the city’s work and directly govern the different city governmental departments/bureaus. We learnt that a local Health China 2030 Action Committee had been established which was led by the MPG of FCG city. The members of this Action Committee are the directors or deputy directors of local governmental departments covering the city’s wider food system and environment. We worked with a designated contact person from a local health authority to liaise with each participant. During the field-work launch ceremony we introduced our project’s objectives, GMB methods, field work plan and the anticipated benefits for FCG city (Fig. 2). We also promoted the concept that the project would be owned by the local stakeholders with the research team playing a supportive role.

#### Cultural and procedural adaptations to the GMB process

We designed and implemented four formal GMB workshops across three different phases in FCG city. The GMB workshops were hosted from 7th June to 1st September 2022. The aim of the phase-1 GMB workshops was to produce a preliminary CLD that demonstrates the common drivers affecting MIAIF in FCG city.

##### Pilot phase-1 GMB workshop

We initially held a hybrid, three-hour, phase-1 GMB pilot workshop in Nanning (capital city of Guangxi Province) to test the feasibility of methodological innovations that allow for mapping inter-related issues on a single CLD, and the ‘hybrid’ format (Fig. 2). The pilot participants were recruited through the research team’s existing networks in Nanning, Guangxi. The participants had no previous experience with this type of participatory research method nor systems thinking approaches. We aimed to have a varied group of participants with an equal gender ratio, and across multiple sectors to reflect the participants of the official GMB workshops more closely. Their feedback informed the methodological elements of the official workshops.

##### Official phase-1 GMB workshop

Phase-1 consisted of two separate workshops. The official workshops each lasted for 3 h, based on the pilot experience. For the first workshop, we invited 16 leaders from 16 governmental departments/bureaus (FCG city’s Propaganda Department, Development and Reform Commission, Education Bureau, Bureau of Industry and Information Technology, Bureau of Finance, Transportation Bureau, Marketing Regulation and Administration Bureau, Medical Security Bureau, Bureau of Agriculture and Rural Affairs, Water Conservancy Bureau, Weather Bureau, Bureau of Urban Administration and Regulation, Tax Bureau, Bureau for Rural Revitalisation, FCG Health Commission and Center for Disease Control and Prevention), which cover the whole local food system. In the participants recruitment process, we ensured that participating officials were working at the same seniority level within the city, which prevented power dynamics issues and promoted open exchanges of views. In the second phase-1 workshop, leaders of six local major food businesses (industry representatives) participated to provide a broader and more complete representation of FCG City’s food system. This was intended to prevent imbalance in power dynamics in the same workshop as the local government has greater authority to make decisions relative to enterprises. The presence of governmental officials may influence the industry representatives’ views on causes of MIAIF. We merged the CLD from each group to produce a comprehensive preliminary CLD.

##### Phase-2 GMB workshop

The phase-2 GMB workshop had the aim of reviewing and adapting the preliminary merged CLD of the phase-1 GMB workshops. We only invited government leaders (n = 13) to the phase-2 GMB workshop in order to maintain a manageable group size and to consolidate the relationships developed in the first phase-1 GMB workshop. The research team tidied up the CLD and improved its visualisation without changing the basic contents co-produced by participants. The results were checked for scientific validity against the latest literature and through consultation with experts from the project’s International Scientific Advisory Board.

##### Direct contribution from the Deputy City Mayor in GMB prior to the final workshop

We added a separate meeting between the phase-2 and phase-3 GMB workshops with the deputy mayor who is responsible for health policies in FCG (Fig. 2). The primary purpose was to obtain feedback on the current version of the CLD, including the themes, and to ensure that the intervention prioritisation process during the final GMB workshop was informed by long-term strategies and priorities of the city.

##### Phase-3 GMB workshop

As per the commonly used GMB process, the final phase-3 GMB workshop was concluded with government leaders from the phase-1 and phase-2 workshops, and front-line professionals/community representatives invited by each governmental department. In this workshop, all participants (n = 18) reviewed and finalised the CLD, and identified and prioritised local intervention targets. Utilising a variety of co-creation activities, including iterative review activities within small groups and the full group, as well as individual anonymous voting for prioritisation of potential intervention targets, we sought to promote inclusive participation while mitigating biased responses. In contrast to the standard GMB, an Action Group was formed after the final GMB workshop. We will report the finalised CLD and prioritized interventions in detail in a separate paper to maintain the methodological focus of this paper.

##### Official establishment of a local Action Group

We ensured the process of forming a multi-department Local Action Group was led by the city government to maximise the sustainability of this systemic, inter-sectoral initiative, and further promote the sense of project ownership among local stakeholders. This can also help to maximise and sustain commitment from group members for optimal impact. An official policy document was issued by the city government which detailed the goals, construction, and responsibilities of the Local Action Group. The formed Local Action Group will cooperate with the project research team and international scientific advisory board to design implementation strategies for systemic nutrition interventions prioritised in the final GMB workshop. Each member department of the Action Group will also jointly implement, monitor and improve the systemic interventions over time with the project research team providing, ongoing scientific/technical support.

##### Other culturally tailored adaptations to foster engagement during the GMB workshops

We made several other adaptions to maximise engagement from all participants in the Chinese setting. We incorporated more open (full group) discussions across the GMB workshops as the participants had limited experience with these types of participatory workshops. Furthermore, both individual- and group-based (collective) prioritisation/voting activities were planned to prevent dominating voices and promote feelings of respect and to encourage participation. We provided each participant with a hand board. This allowed individual participants to express their opinions without the need to speak in front of the group. Within this context, it is socially undesirable to express disagreement in front of others.

### Methodological innovations

**Fig. 3:**
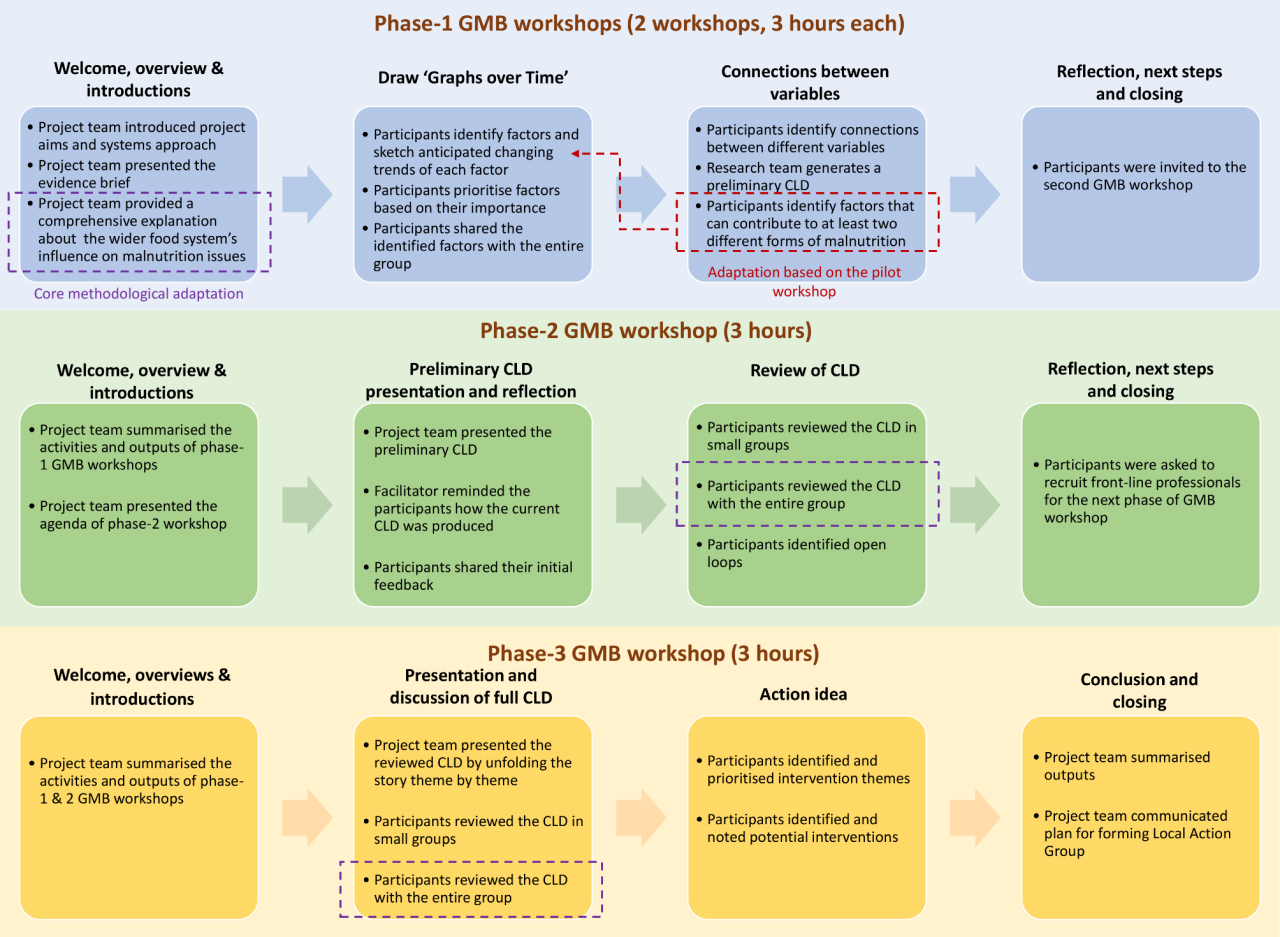
Core activities in the three phases of GMB workshops to tackle MIAIF in FCG city

We also made several major and minor methodological innovations to the standard GMB scripts from Scriptapedia (https://en.wikibooks.org/wiki/Scriptapedia). Figure 3 demonstrates the core activities in the three GMB workshops. A detailed summary of the conducted activities and steps are displayed in Table 1.

**Table 1:** A summary of the steps taken during our GMB workshops

Activity (time) *(Adapted Scriptapedia scripts)*	Description	Steps
Room preparation for each GMB workshop (30 min)	This activity was used to create an inviting and conducive environment for GMB participants before the GMB session begins	Members of both the in-person and online facilitation teams prepared the physical venue, equipment and tools, and Zoom call.
*(Logistics and Room Set Up)*		
**Phase-1 GMB workshops**		
**Welcome, overview and introductions** (30 min)	These activities were used to welcome the participants, warm the room, introduce the project and defined key terms.	The gatekeeper introduced the facilitation team and communicated the team’s expectations for this workshop.
*(Introduction to the project)*		Participants introduced themselves to each other and the team.
		Project team (1) introduced MIAIF using local official prevalence data, (2) defined MIAIF and food systems and prompted participants to think the underlying drivers of malnutrition to shift the focus from individuals to the wider food systems, (3) presented a rationale for using a system approach, outlined project aim, and anticipated benefits of the project to the pilot city.
**Draw ‘Graphs over Time’** (75 min)	These activities were used to engage participants with the problem, describing the reference mode for the workshop and eliciting factors,	Project team (1) engaged participants with the reference mode (the healthiness level of the food environments over time), (2) instructed participants on drawing ‘Graphs Over Time’, and (3) prompted participants to identify factors that can contribute to at least two different forms of malnutrition.
*(Presenting the Reference Mode, Graphs Over Time, Variable Elicitation)*		Participants individually identified factors, drew anticipated changing trends of each factor, and identified factors that can contribute to at least two forms of malnutrition.
		Participants shared, discussed and prioritised factors based on their importance in small groups.
		One representative of each small group shared the identified factors with the full group, and all factors (colour coded to indicate which forms of malnutrition they influence) were entered to STICKE.
**Tea break (10 min)**	This activity was for participants to have a break. It also allowed the local facilitation team to get acquainted with participants and allowed the remote facilitation team to review and act promptly on timing and IT issues.	
**Connections between variables** (60 min)	These activities aimed to facilitate consensus-based group discussion about the connections of identified factors, and to create a preliminary CLD.	Participants identified, discussed and agreed on the connections between different factors.
(Creating Causal Loop Diagram from Connection Circles)		Research team entered collectively agreed connections to STICKE to generate a preliminary CLD
**Reflection, next steps and closing** (5 min)	These activities were used to summarise and close the GMB session and explain the next phase for the participants.	Project team summarised the activities and outputs of the Phase-1 GMB workshop.
*(Next Steps and Closing)*		Project team and gatekeeper expressed their appreciation for the participants’ contributions during the workshop and invited them to the next phase GMB workshop.
**Phase-2 GMB workshop**		
**Welcome, overview and introductions** (10 min)	These activities were used to welcome participants, and to link up the current workshop with the previous workshop.	The gatekeeper introduced the facilitation team and communicated the team’s expectations for this workshop. Participants were invited to re-introduce themselves.
		Project team reflected on Phase-1 workshops by summarising the activities and outputs.
		Project team presented the agenda of Phase-2 workshop.
**Preliminary CLD presentation and reflection** (50 min)	These activities were used to summarise dynamic insights and stories, clarify unclear ideas, capture additional information about model structure, and receive feedback from participants after causal structures have been developed.	Project team presented the preliminary CLD.
*(Modelling Project Community Presentation)*		Facilitator reminded the participants how the current CLD was produced by highlighting the input of the different groups (sectors).
		Project team invited participants to share their initial, general feedback on the existing factors and connections.
**Tea break (10 min)**	This activity aimed to give time for participants to have a break, for local facilitation team to get acquainted with participants, and for remote facilitation team to review and solve IT issues.	
**Review of CLD** (55 min)	These activities were used to continue the modelling reviews, identify (and close) open loops if appropriate, and refine the CLD.	Participants reviewed the CLD in smaller groups of 3-6 people.
*(Model Review)*		Participants reviewed the CLD together as a whole group. The representatives of the small groups were invited to share group feedback (new factors and connections) with the entire group in turns.
		Participants were invited to identify and close open loops if needed by suggesting additional factors.
**Reflection, next steps and closing** (25 min)	These activities were used to explain the next workshop and close the GMB session.	The project team outlined the expectations for the final workshop.
*(Next Steps and Closing)*		Participants were provided with guidance on how to choose and recruit front-line professionals and community representatives for the final GMB workshop.
**Phase-3 GMB workshop**		
**Welcome, overview and introductions** (10 min)	These activities were used to welcome participants, share work that was completed after the last workshop (improving the visual presentation of the CLD and a meeting with the city’s Deputy Mayor), and link up the current workshop with the previous workshops.	The gatekeeper introduced the facilitation team and participants.
		Project team summarised the activities and outputs of Phase-1 and Phase-2 GMB workshops.
		Project team shared the highly supportive feedback on the current CLD and information on the city’s strategic development priorities from the city’s most senior leader for health promotion (Deputy Mayor).
		Project team outlined the agenda of the final workshop.
**Presentation and discussion of the full CLD** (50 min)	These activities were used to disseminate information about the modelling project and elicit feedback from front-line professionals and community-representatives on the current CLD.	Project team presented the developed and reviewed CLD for the enlarged group by unfolding the story theme by theme.
*(Modelling Project Community Presentation, Model Review)*		Participants reviewed the CLD in small groups. Participants reviewed and optimized the CLD as a whole group.
**Tea break** (10 min)	This activity was for the participants to have a break. It also allowed the local facilitation team to get acquainted with participants, and for remote facilitation team to review and act promptly on timing and IT issues.	
**Action idea** (45 min)	These activities were used to identify and prioritise intervention actions after the model has been developed and reviewed.	Participants identified and prioritised intervention themes in small groups and shared ideas with the entire large group.
*(Action Ideas)*		Participants were invited to identify and describe potential intervention ideas with as much detail as possible in writing.
		Participants were invited to prioritise these potential interventions through individual voting and collective discussions until a consensus was made on the ranking of the identified intervention ideas. Participants were asked to prioritise by considering the potential impact and feasibility of potential interventions as well as the city’s long-term objectives.
**Conclusion and closing** (5 min) (Next Steps and Closing)	These activities were used to review today’s workshop, identify the next steps and close the GMB session.	Project team summarised the activities completed at the workshop. Project team communicated the plan for forming an official Local Action Group immediately after the final workshop and expressed their appreciation for the participants’ contributions so far.

#### Mapping and addressing multiple inter-related issues

We chose to implement a step-by-step introductory approach to systems thinking and avoid technical language as previous experiences informed us that participants may find CLDs challenging to build, read and use [31]. In addition to established GMB practices, we also added purposively developed instructions and activities to guide participants’ thinking towards shared drivers of MIAIF. These are described in more detail below. Our protocol was initially tested in a preceding pilot workshop (Fig. 2) (Please see Sect. 2.2.2).

To encourage the participants to think in systems, we included a comprehensive explanation of the wider urban food system in the phase-1 GMB workshop. Corresponding to commonly used GMB processes, we then held a ‘graphs over time’ activity where participants identified determinants and sketched anticipated changing trends of each factor. This exercise allowed our participants to share their understanding of the factors contributing to any form of malnutrition trends within FCG City (5). Following a standard script (called “Graphs Over Time”), the local facilitator provided the participants with a sheet that displayed an empty graph with time on the X-axis (with a vertical line for the present time) and a variable on the Y-axis [6].

To assist the participants with identifying and defining the variables, they were asked about factors they perceived to influence the healthiness level of FCG City’s food environment. Subsequently, as per the standard script, they were instructed to draw two predicted scenarios with and without action respectively. Importantly, we also asked the participants to consider which form(s) of malnutrition were affected by the shared influencing factors. After this activity, we asked the participants to share their identified factors and thoughts with the full group. The facilitation team entered the nominated factors into the online software STICKE. This is a user friendly CLD visual mapping software developed by Deakin University, Australia [32]. The inserted factors were colour-coded differently to clearly communicate which form of malnutrition these influenced. We chose the colours “green”, “purple”, and “red” to respectively denote the factors influencing ‘undernutrition only’, ‘obesity only’, and ‘both under- and over nutrition’. We chose these specific colours as these could be clearly distinguished and displayed on the GMB venue’s screen. The pilot workshop in Nanning showed that the identification of factors that influence both over and under nutrition (or at least two forms of malnutrition) was challenging for the participants in the pilot workshop, as they were not used to thinking in this joined-up way. We therefore prompted the participants of the official, phase-1 workshops to think about this when they identified influencing factors during the drawing activity (Fig. 3).

#### Conducting GMB workshops in a ‘hybrid’ format

**Fig. 4:**
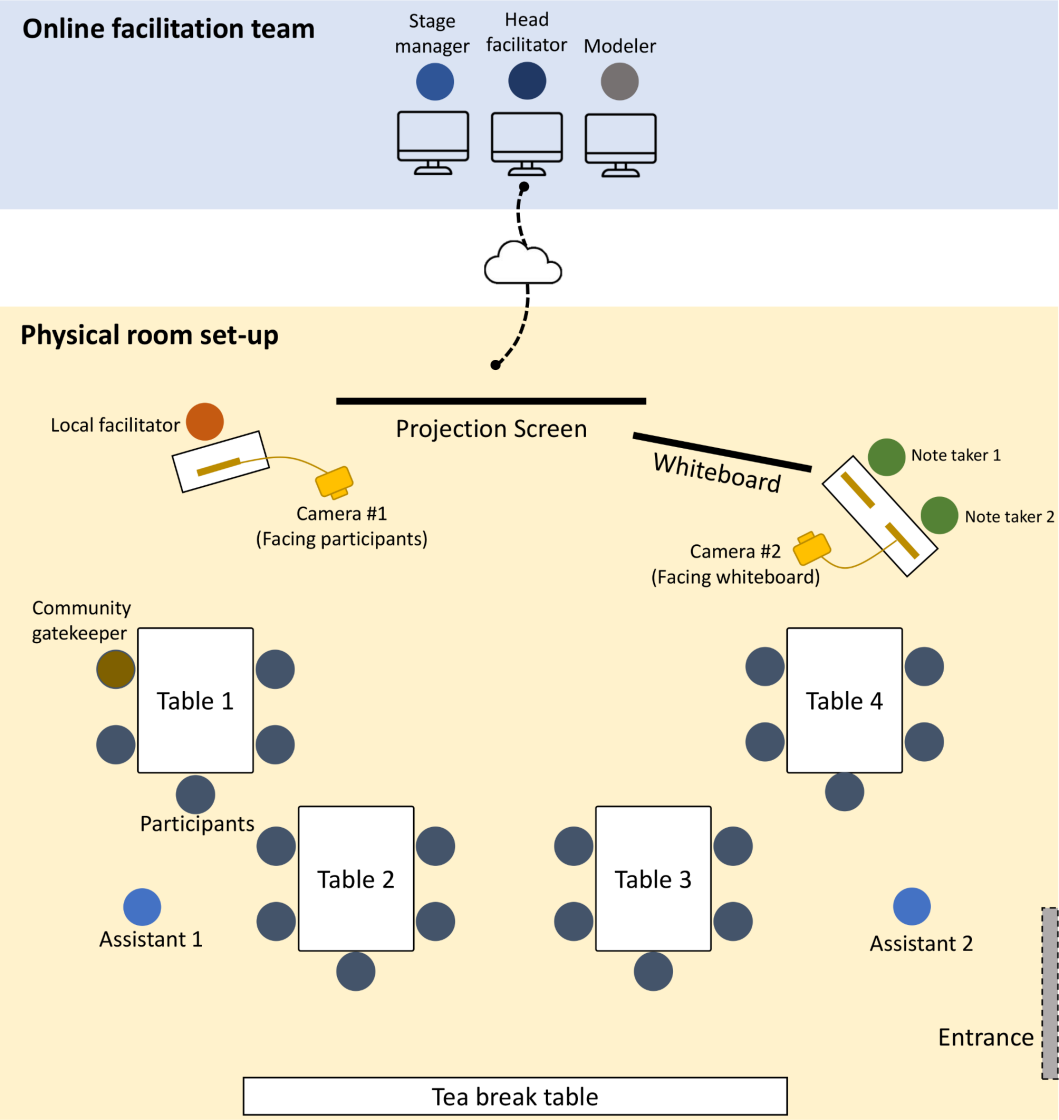
Set-up of a hybrid GMB workshop

Another major innovation is the translation of the in-person GMB protocol to a ‘hybrid’ format. This type of format means that the participants take part in person in one physical venue accompanied by a local facilitating team, while the main facilitating team joining the event online. The professional virtual calling software Zoom was used in the present study for the ‘hybrid’ setting. The ‘hybrid’ workshops were facilitated by two separate teams, one situated in Bristol, UK, and one situated in FCG City. Both teams were led by the head facilitator (BL), who was based in Bristol and is a global health expert specialised in population-level nutrition and physical activity interventions in LMICs and experienced in the practical application of system dynamics methods to nutrition issues. The head facilitator is proficient in both Chinese and English and is familiar with operating in the Chinese setting. The Bristol-based facilitation team also included a modeller who is fluent in both professional English and Chinese and was responsible for constructing the CLD online based on participants’ inputs (Fig. 4). The head facilitator and modeler were complemented by a stage manager (RP), a new role that we purposively introduced to the hybrid process. The stage manager provided technical support during the workshops and was responsible for timekeeping. This allowed the lead facilitator to focus on facilitating the workshop, and most importantly, to efficiently communicate with the participants in China. The FCG team facilitated the workshop at the physical venue in FCG City where all workshop participants were gathered. The physical venue was a conference room in a local hotel which was easily accessible for the participants. The local facilitation team consisted of a table-facilitator (ZH), a community gatekeeper (JL), two on-site assistants to guide and support activities of the participants and 1–2 trained note takers to record the activities and conversations of the participants. The table-facilitator was well-trained for system dynamics and facilitation techniques and is familiar with the local context. The community gatekeeper from the local health authority introduced the two facilitation teams and the participants to warm up the room.

Standard script procedures were taken to prepare the physical room in FCG City. Figure 4 presents the visual physical room set-up. The facilitation team prepared the physical meeting room with fast, stable internet and stereo equipment, a projector and screen, a whiteboard, office stationery, printed documents and refreshment for a tea break. In Bristol, the facilitation team arranged a quiet office space with stable Wi-Fi connection, a multi-tasking laptop, multiple display monitors, wireless laptop devices, and a high quality 1080p webcam to allow for a fluent and time-efficient operation. The Bristol team also printed the named participant seating plan to allow for the facilitator to effectively identify and communicate with the individual participants.

The feasibility of the ‘hybrid’ format, and adapted scripts were tested in the pilot phase-1 workshop in Nanning, Guangxi (see Sect. 2.2.2). This pilot workshop demonstrated the feasibility of the ‘hybrid’ format but identified the need for additional tools to assist participants with reaching consensus when sharing and connecting the identified influencing factor in a groups. We therefore provided each participant in the official GMB workshops with voting tools. These were ‘hand boards’, pairs of traffic light boards in red and green. Red indicates disagreement while green indicates agreement.

## Results

### Cultural adaptations

#### Project engagement activities

The project launch ceremony strengthened the communication with, and engagement of, the city government through multiple mechanisms. First, the attendance of the deputy mayor, officials of the provincial health authority, influential international bodies and experts was featured by national and local media (e.g., satellite television channels and the Chinese government official website) which raised the project’s reputation locally. Furthermore, this event provided an opportunity for the deputy mayor to officially commit support to the project on behalf of the local government.

The subsequent city-level launch ceremony increased the range of awareness and support from leaders of governmental departments/bureaus. Through this event, departmental leaders also became familiarised with systems thinking, and the GMB methods more specifically, which assisted them with participating in the subsequent workshops.

#### Input from the deputy City Mayor

Our meeting with the deputy mayor between the phase-2 and phase-3 GMB workshops promoted a mutual understanding on the project progress, city’s long-term development strategies and prioritised intervention areas between the research team and city government. This shared understanding served as a discussion seed in the phase-3 GMB workshop for departmental leaders and front-line professionals to prioritise and identify potential interventions. It also led to the city government’s commitment to establish a Local Action Group. The official responsibilities of the Local Action Group include implementing, monitoring and improving the co-developed interventions with the aim of maximising the sustainability and impact of the co-developed interventions. The city government’s consent raised awareness and garnered attention from local governmental departments, thereby promoting and expediting the establishment of Local Action Group.

#### Other cultural and operational innovations

The use of the voting tool (a pair of ‘hand boards’) was also beneficial as it promoted active engagement from all workshop participants, including those who felt less comfortable to express disagreement in group discussions verbally. This was particularly helpful in the Chinese setting that values harmony. Moreover, the hand boards provided the online head facilitator with a clear overview of the participants’ opinions. This made the activities and discussions more time efficient.

### Methodological innovations

#### Mapping and prioritising city-wide shared drivers and intervention themes of MIAIF

Generally, the participants were able to carry out all activities in the phase-1 GMB workshops. While some had difficulty with sketching the graphs, the FCG facilitation team was able to support them effectively. This resulted in all participants being able to finish the drawing exercise and small-group discussion within the allocated time.

The adaptations to the standard script allowed the participants (both the participating governmental officials and the food industry representatives) to identify a variety of shared drivers of MIAIF across the food system in FCG city such as environmental pollution, food advertisement, and excessive use of food additives in food production process (detailed results will be presented elsewhere to maintain the methodological focus of this paper). The participants were able to point out the influence of each identified factor on the different form(s) of malnutrition, in contrast to the pilot workshop in Nanning where participants were asked to make these connections towards the end of the phase-1 GMB workshop. Moreover, our utilisation of colour coding for the nominated influencing factors proved to be beneficial as it clearly depicted factors influencing one or multiple forms of MIAIF. The participants also found the colour coding to be a helpful reminder of their co-production process and outputs when there was a prolonged gap between workshops.

#### Operation of ‘hybrid’ GMB workshops

During all workshops, a group of 15 to 20 participants were gathered in the same meeting room within a conference venue. We observed several positive aspects of this hybrid format. The participants were able to pose questions to the head facilitator and receive direct support with exercises from the local facilitation team. Furthermore, the facilitation team at the local venue in FCG city also captured non-verbal cues (e.g., facial expression, eye contact, confirmatory nodding) of the participants. Therefore, the local facilitator was able to assist the online head facilitator with inviting reluctant participants to speak as some of the participants preferred sharing their views in private during the tea break.

The ‘hybrid’ format was both cost- and time-effective due to the absence of intercontinental travel which reduced travel expenditures and was more environmentally friendly.

## Discussion

### Main findings

We described cultural adaptations and multiple methodological innovations to the common GMB process for addressing MIAIF in a culturally novel setting. We demonstrated the implementation of these changes in a Chinese pilot city to support local decision-makers with co-developing city-wide, systemic MIAIF interventions. Our study showed that with methodological, and cultural adaptations, (1) it is feasible to apply GMB for public health nutrition issues in an Asian setting, (2) GMB can be applied to tackle multiple health issues in a joined-up fashion, and (3) well-designed hybrid GMB workshops can cost-effectively assist co development of systemic interventions for MIAIF.

### Strengths and limitations

To the best of our knowledge, this is the first study to develop ways to apply GMB to tackle multiple health issues in a joined-up fashion (in this case MIAIF), the first study to test the feasibility of applying a systems approach to develop nutrition interventions in a LMIC in Asia, and the first study to purposively develop and use a ‘hybrid’ format for GMB.

Another notable strength of our study is that we followed a rigorous process to adapt, pilot and implement GMB workshops in a new research setting. In this process, we carefully considered feedback from participants to iteratively adapt and evaluate the process. Moreover, we engaged a large group of government officials and food industry representatives who represent all parts of the FCG food systems and have the power to change the food systems. Their involvement in the co-creation of the CLD, coupled with valuable input from front-line professionals/community representatives, ensured a diverse and comprehensive interpretation of wider local food system drivers of MIAIF and promoted actionable changes across the local food system. We also promoted the sense of project ownership among the local stakeholders. All of these prepare for rapid and direct translation of the co-production research process to policy implementation. As evidenced in our project, an official Local Action Group has been established at the end of the GMB process to formalize and sustain joint actions from 16 governmental departments of the FCG city to ensure cross-boundary efforts in intervention design, implementation, and evaluation. Furthermore, working across multiple languages was another strength. Local language (Chinese) was used during official workshops with local participants, while international language (English) was used when communicating with research team and international advisory board. This working pattern increased effective engagement and communication with local stakeholders while enabling application of international knowledge transfer. In addition, the ‘hybrid’ design of GMB workshops provided a powerful and cost-effective way to bring global expertise to a locality.

Although our cultural adaptations proved to be successful in a Chinese setting, we anticipate local adaptations are likely to be needed when our hybrid GMB methods are applied to other Asian countries. We recommend local piloting work to be done in other participating countries in the SYSTAM CHINA SEACS consortium project. Moreover, at the current stage of the project, we do not know the implementation and impact of the co-developed interventions (which will be reported elsewhere). However, the established official Local Action Group would allow for long-term inter-departmental monitoring and evaluation. In addition, we only tested the hybrid GMB format in an urban setting with a stable internet connection and high-quality electronic equipment, which may not be available in remote rural areas. Future research may investigate the feasibility of this format in other settings. Finally, the online head facilitator could not always clearly observe the participants’ facial expression or body language. However, the on-site assistants made field notes aiming to capture participants’ behaviours, which helped the online facilitator to better understand participants’ verbal contributions. Future projects that would like to apply a hybrid GMB format may consider the use of high-resolution webcams at both physical and remote venues to further mitigate this issue.

### Findings in relation to the literature and main implications

While previous studies showed GMB to be valuable to understand and address a single, complex public health issue (e.g., childhood obesity) [28, 33], our study demonstrates that it can also be applied to tackle interrelated public health issues such as MIAIF. In our study, our methodological adaptations led to successful mapping of shared drivers of MIAIF in a single CLD. Future research can explore whether our methods could be applied to help identify common drivers of other inter-related issues. Moreover, we included the outcomes of interest (i.e. undernutrition and obesity/diet-related NCDs) as factors in the CLD to support the participants with finding connections between factors, and identify loops and common drivers of MIAIF which has previously been irregularly applied in nutrition- and health-related GMB literature [28, 34, 35]. Future research is required to test whether this approach is also feasible in other cultural contexts/settings.

Regarding the multiple engagement events we added to GMB process in FCG city, while some previous GMB projects might also host multiple meetings prior to the official workshops to engage with local communities or partners, our global scoping review (in press) found no documentations of cultural adaptations or local adaptations to the standard GMB process. In our case, we show that formal launch ceremonies are integral steps in the Chinese setting. Our experiences reinforce that engaging with local initiatives and existing networks is crucial to maximise the success of GMB. Previous studies have also shown that cooperating with local organisations supports participant recruitment and promotes prolonged engagement after the GMB workshops are effective [34]. For example, a recent project by Gerritsen et al. [31] in New Zealand explored declining fruit and vegetable intake among children in West Auckland communities, established a partnership with a local community prevention-based programme (Healthy Familied Waitākere, HFW) to assist the participant recruitment and prolonged action taking. In our case, we conducted GMB workshops with leaders from governmental departments and businesses related to the whole food system in FCG city. Gaining support from a local academic body (e.g., university) or public health organisation was not sufficient as these organisations do not have direct authority to implement long-term regulations and policies. Partnering with governmental agencies to mobilise governmental departments related to food system is essential in contexts such as FCG city. The long-term objectives of FCG city to improve the health of residents also aligned with our research objectives. We therefore cooperated with the Action Committee for the preparations of preceding events and participant recruitment. This way of working proved to be helpful and efficient as it increased the participation of each department and improved communications between the research team and local decision-makers.

We showed that a ‘hybrid’ format is feasible and can be recommended to researchers and policymakers in other Chinese and SEA settings. A growing body of literature shows that fully online GMB workshops are a viable option during crises such as the COVID-19 pandemic when travel is restricted or undesirable [19, 36, 37]. One of the main advantages of the fully online format is that a broad range of researchers, policy makers and community members can gather regardless of their physical locations. Our ‘hybrid’ format mitigated several limitations of fully online workshops including screen fatigue, unstable internet connection, potential IT illiteracy, and limited view of non-verbal communication cues [36]. The tested ‘hybrid’ format also enabled the gathering, and interaction of a large group of participants (15–20 people) at a local venue and promoted a diversity of perspectives. Such a large-scale workshop was common practice for in-person GMB workshops but was reported as a limitation of pure online GMB workshops due to the difficulty in facilitation and communication at an online platform [19, 36]. Moreover, the presence of the FCG in-person facilitation team promotes a wealth of interaction and discussion, capturing of a variety of cues (in addition to the verbal cues), and timely support. The presence of a local facilitator also gave the participants the opportunity to elaborate on their opinions and communicate lingering thoughts to further reflect on the CLD. While the above are also applicable to in-person workshops, with a ‘hybrid’ format, not all facilitators are required to travel which in turn reduces costs, carbon footprint, and allows for workshops to be planned with more flexibility and to be more responsive to unexpected events and issues.

## Conclusions

Our study demonstrated that GMB, with methodological and procedural adaptations, can be applied to co-develop interventions to address MIAIF in an Asian country. The developed ‘hybrid’ GMB format is feasible, cost-effective, and environmentally friendly and is therefore our recommended working model for other Chinese cities, and cities in LMICs in SEA when operating in a low-resource and time-restricted context.

## Data Availability

The raw data supporting the conclusions of this article will be made available by the authors, without undue reservation.

## List of abbreviations

ASEAN: Association of Southeast Asian Nations
CLD: Causal Loop Diagram
FCG: Fang Cheng Gang
GMB: Group Model Building
LMIC: Low- and Middle-Income Countries
MIAIF: Malnutrition In All Its Forms
MPG: Municipal People’s Government
SEA: Southeast Asia
WHO: World Health Organisation
